# Natural History of Nonalcoholic Fatty Liver Disease: Implications for Clinical Practice and an Individualized Approach

**DOI:** 10.1155/2020/9181368

**Published:** 2020-01-21

**Authors:** Ivica Grgurevic, Kristian Podrug, Ivana Mikolasevic, Michal Kukla, Anita Madir, Emmanuel A. Tsochatzis

**Affiliations:** ^1^Department of Gastroenterology, Hepatology and Clinical Nutrition, University Hospital Dubrava, University of Zagreb School of Medicine, Zagreb, Croatia; ^2^Department of Gastroenterology and Hepatology, University Hospital Centre Split, Split, Croatia; ^3^Department of Gastroenterology and Hepatology, University Hospital Centre Rijeka, University of Rijeka School of Medicine, Rijeka, Croatia; ^4^Department of Gastroenterology and Hepatology, School of Medicine in Katowice, Medical University of Silesia, Katowice, Poland; ^5^UCL Institute for Liver and Digestive Health, Royal Free Hospital, London, UK

## Abstract

Nonalcoholic fatty liver disease (NAFLD) is becoming the most prevalent liver disease worldwide, associated with epidemics of overweight and resulting metabolic syndrome (MetS). Around 20–30% of patients with NAFLD develop progressive liver fibrosis, which is the most important predictor of liver-related and overall morbidity and mortality. In contrast to classical understanding, no significant association has been demonstrated between the inflammatory component of NAFLD, i.e., nonalcoholic steatohepatitis (NASH), and the adverse clinical outcomes. Older age (>50 years) and presence of type 2 diabetes mellitus, in addition to some genetic variants, are most consistently reported indicators of increased risk of having liver fibrosis. However, critical driving force for the progression of fibrosis and risk factors for this have still not been fully elucidated. Apart from the genetic profile, gut dysbiosis, weight gain, worsening of insulin resistance, and worsening of liver steatosis represent candidate factors associated with unfavourable development of liver disease. Cardiovascular events, extrahepatic malignancies, and liver-related deaths are the leading causes of mortality in NAFLD. As patients with advanced fibrosis are under highest risk of adverse clinical outcomes, efforts should be made to recognize individuals under risk and rule out the presence of this stage of fibrosis, preferably by using simple noninvasive tools. This process should start at the primary care level by using validated biochemical tests, followed by direct serum tests for fibrosis or elastography in the remaining patients. Patients with advanced fibrosis should be referred to hepatologists for aggressive lifestyle modification and correction of the components of MetS, and cirrhotic patients should be screened for hepatocellular carcinoma and oesophageal varices.

## 1. Introduction

Nonalcoholic fatty liver disease (NAFLD) is now considered the most prevalent chronic liver disease (CLD) worldwide [1, 2]. It is expected to become the most common cause of end-stage liver disease (i.e., cirrhosis and hepatocellular carcinoma) in the near future and, consequently, the most common indication for liver transplantation (LT) [3, 4]. The prevalence of NAFLD goes hand by hand with the prevalence of overweight and obesity, as well as metabolic syndrome (MetS), and due to its multisystemic effect, this combination represents the most serious health threat responsible for increasing number of cardiovascular, oncologic, and liver-related morbidity and mortality [1, 2, 5–7]. Over the last two decades, significant improvements have been achieved in understanding the natural history of NAFLD, which was important to understand the clinical behaviour of the disease [2]. This in turn enabled more precise risk stratification and the development of rational diagnostic pathways with the final aim of preventing liver-related and other complications [8]. In this review, we intended to present the current knowledge on the natural history of NAFLD and its implications for a rational and more individualized approach to this condition, harbouring features of global epidemics.

## 2. Epidemiology of NAFLD

The prevalence of NAFLD in the general population varies from 13.48% in Africa to 30.45% in South America and 31.79% in the Middle East [1, 2]. The prevalence in Europe is 23.71% and in the United States 24.1% [2]. The prevalence in the United States differs among ethnic groups, the highest being in Hispanic Americans (29%), and even by the country of origin (Mexican Americans 33% and Dominicans 16%) [2]. NAFLD is defined as the presence of >5% of liver steatosis in the absence of other causes of steatosis and CLD (chronic viral hepatitis, autoimmune and other metabolic liver diseases, and the use of medications that can induce steatosis) in the absence of significant alcohol consumption (>21 drinks/week in men and >14 drinks/week in women) [2]. The presence of simple steatosis is defined as nonalcoholic fatty liver (NAFL), whereas nonalcoholic steatohepatitis (NASH) is a more aggressive form of NAFLD that includes a histological presentation of steatosis, ballooning, and lobular inflammation that leads to fibrosis, cirrhosis, and hepatocellular carcinoma (HCC) [2, 3]. Recent data revealed that almost 15–20% of HCCs occur in NAFLD patients without cirrhosis [2, 3].

NAFLD is closely related to metabolic syndrome (MetS) and its individual components: diabetes mellitus type 2 (T2DM), arterial hypertension, dyslipidaemia, and obesity [2, 5]. Actually, NAFLD has been recognized as the liver manifestation of MetS [5–7]. Therefore, the majority of NAFLD patients have metabolic comorbidities, and a small proportion of NAFLD patients have “lean” NAFLD [2]. The prevalence of lean NAFLD is 7% in the US and up to 25% in some Asian countries [2, 4]. However, the majority of NAFLD cases are related to MetS and its individual components [4, 5]. According to a meta-analysis, the prevalence of NAFLD, NASH, and advanced fibrosis (*F* ≥ 3 according to the METAVIR scoring system) in T2DM patients was 57.8%, 65.26%, and 15.05%, respectively [2, 4]. In addition, the overall prevalence of dyslipidaemia among NAFLD and NASH patients was 69.16% and 72.13%, while the overall prevalence of hypertension was 39.34% and 67.97%, respectively [2]. Finally, the prevalence of MetS among NAFLD and NASH patients was 41% and 71%, respectively [2, 5]. Regarding the association between NAFLD and obesity, the majority of morbidly obese patients undergoing bariatric surgery have NAFLD, 20% to 30% of them have NASH, and 10% have advanced fibrosis [9]. The high prevalence of MetS and its individual components in NAFLD patients suggests a high risk for CVD in addition to liver-related morbidity, while an increased risk of other extrahepatic chronic diseases (chronic kidney disease, T2DM, colorectal cancer, etc.) has been demonstrated as well [2, 6, 7, 10].

## 3. Natural History and Predictors of Mortality in NAFLD

The accumulation of new scientific knowledge has provided better insights into the natural course of NAFLD. Two European studies demonstrated the higher mortality of NAFLD patients relative to the general population, whereas mortality was lower in comparison to patients with alcoholic liver disease (ALD) and hepatitis B and C [11, 12]. With an average follow-up of 13.7 and 28 years, the mortality of NAFLD patients reached 22% and 40%, respectively, which was 37.5% and 69% higher than in the general population [12]. The three most common causes of mortality for patients with NAFLD were cardiovascular diseases (30% to 61.5% of cases), followed by extrahepatic malignancies (19% to 28%) and liver-related deaths (7.7% to 19%) [11, 12]. In both studies, when compared to the general population, mortality was significantly higher only in patients with NASH and not in those with isolated steatosis [11, 12]. In the cohort from the US, 131 NAFLD patients were followed for an average of 18.5 years: overall mortality was 59.5%, irrespective of the presence of NASH. Likewise, the main causes of death were coronary disease (28.2%), extrahepatic malignancies (17.9%), and liver-related complications (15.4%). However, liver-related mortality was significantly higher in patients with NASH compared to non-NASH counterparts (17.5% vs. 2.7%; *P*=0.0048), as well as in diabetics, elderly patients, and those with reduced albumin at baseline [13].

It should be noted that, in these earlier studies, all patients with NASH were analysed as a cohort with no specific distinction with regard to the presence of fibrosis versus isolated steatosis; hence, most of them had some degree of fibrosis. It is therefore not surprising that NASH was considered as a risk factor for a worse outcome. Nevertheless, even at that time, Ekstedt and colleagues demonstrated that the absence of periportal fibrosis at baseline liver biopsy had a 100% negative predictive value (NPV) for the development of liver-related complications [11]. Subsequent studies have separately analysed the individual histological categories within NAFLD, thus enabling a more detailed risk stratification. In a multicentre study involving 619 patients with histologically verified NAFLD who were followed for an average of 12.6 years, the overall mortality was 33.2%, of which 38.3% was due to cardiovascular diseases, 18.7% was due to extrahepatic malignancies, and 8.8% was due to liver-related complications [14]. Among the analysed histological categories, only fibrosis and not steatosis, nor the presence of liver inflammation (i.e., NASH), was associated with overall and liver-related mortality [14]. Independent predictors of overall mortality were higher stages of fibrosis, starting already from stage 1 relative to stage 0, older age, diabetes, and smoking, while statin administration had a protective effect [14]. The single independent predictor of liver-related mortality was the stage of fibrosis, starting from stage F2 [14]. The effect and interaction of the stage of fibrosis and the presence of NASH on survival without liver-related complications have been analysed separately [14]. Survival was significantly higher in patients without fibrosis than in the patients with fibrosis, regardless of the presence of NASH [14]. Similar conclusions were reached by the authors of another study that analysed the survival of a cohort of 229 patients with NAFLD, followed for an average of 26.4 years. In this cohort, significantly higher mortality relative to the general population was observed only in patients with F3 and F4 stages of liver fibrosis regardless of the presence of NASH, whereas mortality was not increased in patients with NASH who had lower stages (F0 to F1) of fibrosis [15]. In another large Swedish study, 646 patients with NAFLD were followed for an average of 20 years [16]. The presence of NASH was not associated with liver-related or overall mortality. Following the adjustments for age, sex, and the presence of diabetes, the risk of overall mortality was only increased in patients with F3 and F4 stages of fibrosis relative to NAFLD patients without fibrosis [16]. The presence of NASH did not influence the prognostic impact of fibrosis, whereas the stage of fibrosis significantly affected the prognosis in patients with NASH [16]. The same results were obtained for liver-related outcomes defined as the decompensation of cirrhosis or the development of HCC. The time within which the first 10% of patients from each stage of fibrosis would have developed severe liver diseases was estimated to be 2.3 years for patients with stage F3, 9.3 years for stage F2, and 22 to 26 years for stage F0-1 [16]. According to the meta-analysis, which included five studies with a total of 1,495 NAFLD patients and 17,452 patient-years of follow-up, an exponential increase in overall mortality and especially liver-related mortality was observed with worsening stages of fibrosis [17]. The overall mortality increased significantly already at stage F1 relative to F0, while liver-related mortality increased only starting at stage F2 [17]. The main limitation of this meta-analysis is the lack of data on cofounders and as such, it was not possible to adjust the results according to age, sex, and comorbidities (e.g., T2DM) [17]. It is interesting to note that, in the large multinational cohort study of 458 NAFLD patients with advanced fibrosis (F3 and F4) at baseline biopsy, those with F3 had more vascular events and nonliver cancers during the mean follow-up period of 5.5 years, whereas patients with compensated cirrhosis more frequently developed liver decompensation, HCC, and liver-related death. Liver-related events were associated with the presence of cirrhosis and mild steatosis (<33% fatty transformed hepatocytes), and history of moderate alcohol consumption contributed to these outcomes only in patients with cirrhosis and not F3 fibrosis [18].

The relationship between T2DM and NAFLD is of particular interest, as it shows a bidirectional interaction. T2DM is found in about a quarter of patients with NAFLD, while NAFLD is found in about three quarters of patients with T2DM [2, 19]. As previously mentioned, mortality was significantly increased in patients with NAFLD and T2DM [19]. The same was observed in T2DM patients with NAFLD, for whom the risk of mortality was about twofold higher than in those without NAFLD (two studies with a total of 2,350 patients with T2DM monitored for an average of 6.5 and 11 years) [20, 21]. However, in one abstract by an American group of authors, the mortality in patients with T2DM who had NAFLD was not higher relative to those without NAFLD [22]. Although it seems intuitive, the screening for NAFLD in patients with T2DM has not proven to be effective in terms of cost-benefit analysis, primarily due to the limited potential and the side effects of available medications for NAFLD, as well as the lack of reliability of noninvasive diagnostic methods that still need to be examined in the population with T2DM, which should also be taken into account [23–25].

### 3.1. Fibrosis Progression in NAFLD

Fibrosis progression does not occur in all patients with NAFLD and not at the same rate. Data on the fibrosis progression in NAFLD are based on a small number of studies in which a paired biopsy was performed at follow-up. In a meta-analysis involving 11 studies that included 411 patients with an average 14-year interval between two liver biopsies, interesting data were obtained [26]. Fibrosis progression was observed in 36% of patients, with no difference in the proportion of progressors between patients with isolated steatosis and those with NASH [26]. Accordingly, fibrosis progresses regardless of NASH, and isolated steatosis does not exclude the possibility of progressive fibrosis development [26]. However, the rate of progression of fibrosis was higher in patients with NASH (about seven years for one histological stage of fibrosis) relative to isolated steatosis (14 years for one stage of fibrosis) [26]. In the group of progressors, about 20% developed severe fibrosis (F3 to F4) very quickly (six years on average) regardless of the presence of NASH [26]. In the multivariate analysis, factors associated with rapid progression were hypertension and a low baseline AST/ALT ratio [26]. Limitation to this study should be acknowledged due to selection bias as follow-up biopsies were not performed per protocol but were performed as a response to clinical need or suspicion of the disease progression. Similar results were presented in a recent study conducted with 60 NAFLD patients with an interval of 8.4 years between paired liver biopsies [27]. The progression of fibrosis was observed in 43% of patients, regardless of the presence of NASH or isolated steatosis at the baseline biopsy, and there was also no difference in the rate of progression between the groups (about seven years for one stage of fibrosis in NASH and about 10 years in patients with isolated steatosis) [27].

Recently, data from the prospective phase 2b placebo-controlled trial of simtuzumab for the treatment of NAFLD patients (*N* = 475) with F3 and F4 fibrosis who had baseline and follow-up biopsies were presented. Since the investigated compound failed to show, the efficacy trial was terminated after 96 weeks, and patients from all treatment groups were then analysed together. Progression to cirrhosis was observed in 20% of F3 patients, and liver-related events in 19% of patients with baseline cirrhosis. Both outcomes were related to the amount of fibrosis at baseline and at follow-up (quantified by histology and serum markers of fibrosis), but not to necroinflamatory activity as expressed by the NAFLD Activity Score [28].

The risk of fibrosis is to some extent genetically determined, and several genetic variants (single nucleotide polymorphisms, SNPs) associated with the progression of fibrosis have been identified [29]. Patatin-like phospholipase domain-containing protein 3 (PNPLA3, also known as adiponutrin) and transmembrane 6 superfamily member 2 (TM6SF2) have been most thoroughly evaluated [29]. The replacement of the isoleucine with methionine at the 148 codon (I148M) of PNPLA3 results in decreased hydrolysis of triglycerides in hepatocytes, while the E167K variant of TM6SF2 (change of glutamate to lysine at codon 167) leads to decreased VLDL secretion from the liver [29]. Both polymorphisms are associated with a higher risk of developing steatosis, NASH, cirrhosis, and HCC [29]. According to the results of a meta-analysis, carriers of the I148M PNPLA3 polymorphism (rs738409C/G), i.e., GG homozygotes, have 73% more fat in the liver and a 3.2-fold higher risk of developing fibrosis than CC homozygotes [29]. Recently, the genetic variant (*rs72613567:TA*) in hydroxysteroid 17-beta dehydrogenase 13 (HSD17B13) was shown to be associated with decreased risk of NASH and liver fibrosis. This polymorphism leads to decreased expression of the HSD17B13 protein in hepatocytes and reduced enzymatic activity against some biological compounds such as leukotriene B4, which is involved in lipid-mediated inflammation, as well as against oestradiol [30]. In addition to genetics and the indicators at the beginning of the clinical evaluation of a patient, features indicating a higher risk of fibrosis progression during the monitoring period have been identified. They comprise elevated ALT and AST, the worsening of insulin resistance, weight gain of at least 5 kg, decreased platelets, and the worsening of liver steatosis relative to the initial biopsy [11]. In another study, the development of diabetes and a higher FIB-4 index during the follow-up period were also identified as significant [31].

### 3.2. Fibrogenesis in NAFLD: NASH vs. Other Mechanisms

Since NAFLD/NASH is a complex disorder of multifactorial aetiology, based on multiple parallel hits, the critical pathophysiological mechanisms responsible for fibrosis development and progression are still not fully explained [32]. The main pathways involved in fibrogenesis are associated with insulin resistance, lipotoxicity resulting from free fatty acid (FFA) overload and their derivatives, environmental factors such as diet, obesity, and microbiota, genetics, endoplasmic reticulum stress, mitochondrial dysfunction, hypoxia, apoptosis, and ultimately, hepatic stellate cell (HSC) activation [32–35]. It is interesting to note that, in some cases, fibrosis is not NASH dependent and inflammation is not a key mechanism of fibrosis development [27].

Adipose tissue lipolysis, de novo lipogenesis from glucose and fructose and dietary fat are the main sources of FFAs stored in the liver. The overflow of FFAs results in the depletion of hepatocytes' ability to produce triglycerides (TGs) [32]. Excessive FFAs are transformed into lipotoxic agents, which impair the endoplasmic reticulum and mitochondria, evoke oxidative stress, activate inflammasomes, and potentiate apoptosis [32, 33]. Microsomal FFA metabolism induces ROS production in the liver [34]. Oxidized FFAs can also catalyse lipid peroxidation reactions that are directly cytotoxic [35]. Pointing to the abovementioned mechanisms, the impairment of FFA disposal or increased inflow to the liver alongside the depletion of their detoxification results in fibrosis progression independent of NASH [32].

Gut dysbiosis has been suggested as an additional factor for the development and progression of NAFLD [36, 37]. An elevated ratio of Gram-negative to Gram-positive bacteria and *Firmicutes* to *Bacteroidetes* with an upregulated number of mucous-degrading bacteria impairs the gut barrier NAFLD [38]. This in turn leads to the increased translocation of bacterial fragments and endotoxin absorption to the portal blood flow that finally enters the liver [39]. These compounds activate signalling pathways in the liver depending on nuclear factor *κ*B (NF-*κ*B) and pattern recognition receptors (PRRs) either localized on cell membrane surface Toll-like receptors (TLRs) or localized in cytosol NOD-like receptors (NLRs). NLRs are linked to inflammasomes, protein complexes which when activated stimulate cell apoptosis, and the release of proinflammatory cytokines from inflammatory cells. Very high inflammasome activity was found in NAFLD patients and was associated with insulin resistance, high levels of FFAs, the overproduction of leptin, and the downregulation of adiponectin synthesis [40]. Inflammasomes and absorbed lipopolysaccharides (LPSs) may directly affect HSCs and macrophages (Kupffer cells) by stimulating the production of smooth muscle actin, transforming growth factor *β*1 (TGF *β*1), and collagen fibres, which promotes the development and progression of fibrosis [41]. Even a small amount of intestinal endotoxins may have an effect on increased sensitivity to leptin, exacerbating fibrosis progression [41]. Gut microbiota exerts significant interaction with bile acids (BA) influencing their chemical structure and composition. This in turn leads to altered BA signalling, potentially contributing to the development of liver fibrosis [42].

Oxidative stress, lipid overload of hepatocytes, and the impaired function of mitochondria lead to an energy imbalance and hypoxia [33, 34]. Hypoxia stimulates neoangiogenesis, which might be observed even in bland steatosis and seems to be independent of NASH [43]. The development of fibrosis is preceded by angiogenesis, but in later stages, angiogenesis strictly correlates with the severity of fibrosis. NASH in severely obese patients had no influence on the hepatic expression of angiogenic factors [43, 44].

NAFLD is associated with an improper adipokine profile with increased levels of leptin and a decreased concentration of adiponectin [45–47]. Leptin promotes fibrogenesis indirectly through the activation of Kupffer cells and sinusoidal endothelial cells (SECs) through the upregulation of TGF*β*1 production and directly by the activation of HSCs [48–51]. Additionally, leptin induces proliferation and inhibits the apoptosis of HSCs [52].

Adiponectin has a hepatoprotective and antifibrogenic effect in cases of liver injury and protects against liver steatosis [48, 51]. This effect is independent of its metabolic action and is associated with the modulation of HSCs, which express both adiponectin receptors [53]. Adiponectin activation of AMPK disrupts leptin-mediated hepatic fibrosis by upregulating suppressors of cytokine signalling 3 (SOCS-3) [54].

Hyperinsulinaemia promotes profibrogenic signals in HSCs, either directly or as a cofactor of TGF-*β*1 [55]. In addition, hyperglycaemia, which is commonly observed in NAFLD patients, determines the occurrence of the nonenzymatic glycation and oxidation of proteins and lipids, resulting in the formation of advanced glycation end products (AGEs). HSCs express a receptor for AGEs and undergo activation when exposed in vitro to glyceraldehyde-derived AGEs. Moreover, AGEs may activate fibrogenesis through the modulation of TNF*α*-converting enzyme activity [56, 57]. These results explain why the degree of insulin resistance IR is related to disease severity and the fact that NAFLD patients with coexisting T2DM exhibit more progressive disease and faster fibrosis progression.

## 4. How to Recognize the Risk of Liver Fibrosis in NAFLD

The question is how to identify patients with NAFLD who have a higher risk of fibrosis on the basis of simple clinical features. Studies that examined clinical risk (mostly cross-sectional studies with only baseline liver biopsy) identified older age (usually >50 years), higher BMI (>28 to 30 kg/m^2^), T2DM, NAFLD Fibrosis score (NFS), and FIB-4 score as the indicators for the presence of fibrosis in NAFLD patients [14, 30, 58]. The prevalence of liver fibrosis in the general population has been investigated using noninvasive methods [59]. The Rotterdam study evaluated 3,041 people ≥45 years of age from the general population without a history of chronic liver disease [60]. All of them underwent transient elastography (TE) of the liver. Liver stiffness of ≥8 kPa was considered clinically significant, and liver steatosis was determined by ultrasound [60]. Clinically significant fibrosis was found in 5.6% of subjects, with a significant association with liver steatosis and T2DM [60]. Off note, given the modest predictive value of TE for advanced fibrosis (with cutoff values generated at tertiary care centres), the true prevalence of clinically significant fibrosis in general population is probably even lower [59]. Accordingly, the simple clinical risk profile for fibrosis is constituted by persons >50 years of age, overweight, and diabetic. By using simple noninvasive diagnostic tools, additional risk indicators including fatty liver was determined by ultrasound and elevated values of biochemical indicators such as FIB-4 and NFS [9, 24, 60, 61]. The prevalence of advanced fibrosis (*F* ≥ 3) was reported to be 15.05% in T2DM patients with NAFLD [2] and 10% in obese patients undergoing bariatric surgery [9]. Since central obesity and diabetes are both components of MetS, it is plausible to expect an incremental rise of risk for liver fibrosis in patients with more components of MetS. Indeed, T2DM increases the adjusted risk for significant to severe fibrosis by 25.41%, while NAFLD patients with T2DM and hypertension have a 26.32% risk of significant to advanced fibrosis [5, 62]. More recently, Younossi et al. have demonstrated that MetS in NAFLD patients is strongly associated with increased cardiovascular, liver-related, and all-cause mortality [63]. According to these data and expert consensus, NAFLD patients with T2DM and other MetS components who have increased liver enzyme levels are at the highest risk for significant and advanced fibrosis and should be considered for liver biopsy [4, 63].

## 5. Implications for Clinical Practice

### 5.1. Should We Search for NAFLD?

The risk of having fatty liver obviously goes hand in hand with the epidemiology of overweight/obesity, and it is frequently observed in patients with one or more components of MetS [1, 5]. Fatty liver can be easily diagnosed by ultrasound, and it is a widely available, noninvasive, and cheap procedure. In areas where ultrasound is not available, simple biochemical indices might be used instead (for example, the fatty liver index) [61, 64]. However, to actively search for fatty liver is a very contentious issue. As already pointed out from the results of the Rotterdam study, significant fibrosis was associated with the presence of fatty liver especially in combination with T2DM. On the other hand, most guidelines do not recommend screening for NAFLD. It might be worth exploring if people at risk for NAFLD should be screened for fibrosis, but this needs to be proved by further data.

### 5.2. What to Search for in a Patient Diagnosed with NAFLD?

The results of the quoted studies have important implications for clinical practice. Firstly, the most important prognostic category in patients with NAFLD is the stage of liver fibrosis, as it determines the risk of developing liver-related complications and overall mortality [15]. In that regard, patients with an established diagnosis should be tested for the presence of advanced fibrosis as they are associated with the risk of overall and liver-related mortality [15, 16, 64, 65]. Patients with advanced fibrosis should be monitored by a hepatologist [8, 15]. Patients who have been diagnosed with stage F2 fibrosis should also be monitored by a hepatologist particularly in the presence of metabolic comorbidities such as obesity and diabetes [8, 16]. Patients in stages F0 to F1 are not expected to develop complications of liver diseases over a period of about 20 years and may continue to be monitored at the primary care level [16, 65]. Secondly, diabetes worsens the prognosis of NAFLD, whereas some disagreement exists about the effect of NAFLD on mortality in patients with diabetes [20–22]. Therefore, an important clinical goal is the adequate regulation of diabetes. Thirdly, NASH without the presence of fibrosis does not significantly affect prognosis, so diagnostic tests to determine its presence are not of critical clinical importance (with the exception of biopsy, all noninvasive NASH tests have not shown sufficient diagnostic reliability) [26, 27, 57, 62]. The regression of fibrosis, rather than the curing of NASH, appears to be a key goal in treating patients with NAFLD [32]. Fourthly, the degree of steatosis also does not indicate the risk of fibrosis, nor is it associated with clinical outcomes in terms of overall mortality or liver-related mortality, and as such, it does not constitute relevant prognostic data. However, since the worsening of steatosis during follow-up may be associated with the progression of fibrosis [11] and the reduction of steatosis may be a measurable parameter for the success of dietary measures, it seems that the quantification of steatosis at baseline and during follow-up may be a useful parameter, although additional studies are required to support this claim.

Finally, since NAFLD is closely related to insulin resistance, MetS, and its individual components (T2DM dyslipidaemia, obesity, and arterial hypertension), all NAFLD patients should be evaluated for these conditions by performing simple anthropometric measurements and simple laboratory tests [8, 66]. In addition, it has become clear that NAFLD is not only a “liver disease” but also a risk factor for many other extrahepatic diseases, including cardiovascular diseases (CVD), chronic kidney disease (CKD), T2DM, and colorectal cancer [7]. According to these data, physicians who manage NAFLD patients should recognize and evaluate extrahepatic manifestations of NAFLD and should not only focus on liver disease [6, 7, 67, 68].

### 5.3. How Should Liver Disease and Associated Extrahepatic Conditions in NAFLD Be Diagnosed?

**Figure 1:**
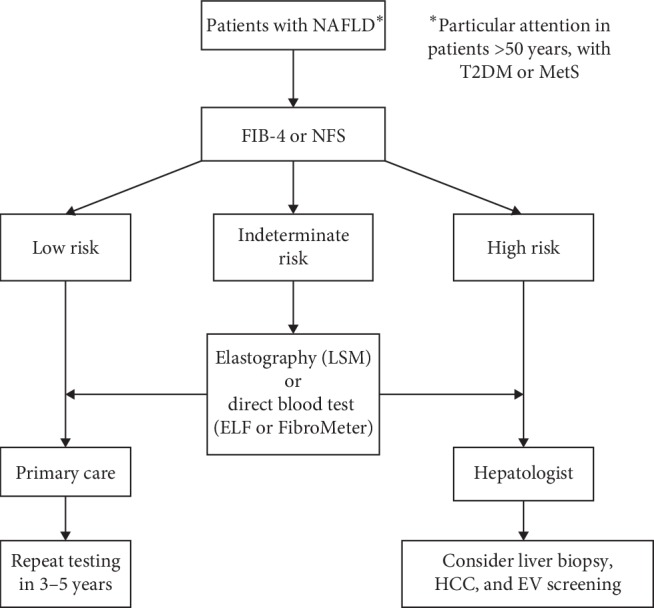
Proposed diagnostic algorithm to stratify patients with nonalcoholic fatty liver disease (NAFLD) according to the risk of having advanced (*F* ≥ 3) liver fibrosis. Diagnostic process begins by using first tier testing-nonproprietary algorithm (FIB4 or NFS) from available simple blood tests and demographic data. Those with low risk need no further testing and might be followed in primary care (general practitioners) with repeated risk assessment at 3–5 years. Those with high risk require immediate referral to secondary care (hepatologist). Patients with indeterminate risk should proceed to second-tier testing, liver stiffness measurement (LSM) by elastography, or direct blood tests for fibrosis by patented algorithms (such as ELF or FibroMeter). ELF = enhanced liver fibrosis score; EV = oesophageal varices; HCC = hepatocellular carcinoma; MetS = metabolic syndrome; NFS = nonalcoholic fatty liver disease fibrosis score; T2DM = type 2 diabetes mellitus.

Given the epidemiological characteristics, i.e., the prevalence of NAFLD and the fact that the vast majority of patients will never develop severe liver disease, it is neither realistic nor necessary to perform liver biopsy in all patients. A diagnostic algorithm for NAFLD should be simple, using preferably noninvasive tools that are widely available and provide reliable results [8, 61, 64]. The goal of noninvasive methods should be to reliably quantify the amount of liver fibrosis [15, 17]. Advanced fibrosis, i.e., stages F3 and F4, should be ruled out first by a simple biochemical test (e.g., FIB-4 or NFS) at the primary care level [64, 65]. These tests proved better in terms of diagnostic performance for advanced fibrosis as compared to other simple biochemical tests such as APRI or BARD [69, 70]. Such patients may continue to be treated by a family physician with the correction of components of MetS and a reevaluation of the stage of liver fibrosis in three to five years [64, 65]. Patients with a high or intermediate risk of severe fibrosis based on the results of noninvasive biochemical tests should be referred to a second line of testing, which may include another biochemical test (preferably direct tests measuring extracellular matrix components, such as the ELF test of FibroMeter) or liver elastography (TE being the best validated; LSM values <8 kPa exclude significant fibrosis, LSM >9.5 to 10 kPa indicate severe fibrosis or compensated advanced chronic liver disease, while values of >15 kPa suggest cirrhosis [61, 71, 72]). Patients in whom the second line of tests confirms advanced fibrosis should be referred to a hepatologist in order to reliably assess the presence of cirrhosis and portal hypertension, as these require further specific investigations that include screening for the presence of oesophageal varices and HCC [73]. Since noninvasive tests have a modest positive predictive value for cirrhosis (about 50 to 70%), patients who have been diagnosed with cirrhosis based on noninvasive tests usually need a liver biopsy to reliably determine the stage of fibrosis, as well as to assess the presence of other histological components [8, 74]. As explained in the previous paragraph, it may be useful to quantify steatosis at baseline and during the follow-up period, although solid scientific evidence is still lacking. The best validated noninvasive measure for this purpose is the controlled attenuation parameter (CAP) coupled with TE [61, 64]. Proposed algorithm for risk stratification in patients with NAFLD is depicted in Figure 1. Patients with NAFLD have a two- to fivefold higher risk for the development of T2DM, and accordingly, screening for T2DM in NAFLD patients should be implemented by periodically performing simple laboratory tests (fasting or random blood glucose, HbA1c, and standardized 75 g OGTT in high-risk groups) [6, 8, 63]. NAFLD is associated with an enhanced risk of CVD and its progression [10, 67]. The age and the presence of MetS and its individual components as well as the presence of significant fibrosis are risk factors for CVD in NAFLD patients; these patients should therefore be referred to a cardiologist [75]. The CVD risk in this subgroup of NAFLD patients can be assessed by dobutamine stress ECHO, CT coronary angiography, and/or coronary angiography, which is the gold standard [75]. On the other hand, there are insufficient data to recommend the screening of NAFLD patients with advanced liver disease who are not candidates for LT, as well as those without significant fibrosis. Asymptomatic, low-risk patients should be evaluated for traditional CVD risk factors (i.e., MetS components). Methods used to estimate CVD risk in the general population, such as the Framingham Risk Score, need to be validated in patients with NAFLD [10, 75]. Consequently, the current data are insufficient regarding the optimal screening strategy for asymptomatic NAFLD patients, and further studies are needed [75]. Since the association between NAFLD and the development of chronic kidney disease (CKD) has been demonstrated as well, NAFLD patients could benefit from annual screening for CKD [68]. This may include the analysis of simple laboratory parameters that are available in every day clinical practice (such as the determination of serum creatinine and albuminuria) [65, 68].

### 5.4. Who Should Be Screened for HCC?

NAFLD is increasingly becoming an aetiological factor for HCC, and it is likely that, in the near future, NAFLD will become the leading cause of HCC [76]. Although HCC predominantly develops against a background of liver cirrhosis, 15 to 20% of HCC in NAFLD occur in the noncirrhotic liver [3, 76]. Given the high prevalence of NAFLD in the general population, this observation calls for increased attention. Effective screening programmes are currently lacking in part because current knowledge does not allow for the precise stratification of cancer risk in NAFLD patients [76]. However, according to a large American study, NAFLD, age >65 years, T2DM, and Hispano race have been identified as independent predictors of HCC risk in the general population [77]. In NAFLD patients, cirrhosis, age >65 years, male sex, and Hispano race were associated with a higher incidence of HCC. Nevertheless, cirrhosis was demonstrated to be the main risk factor because the incidence of HCC in patients with NAFLD cirrhosis was 13.55 per 1,000 patient-years, compared to only 0.04 per 1000 patient-years in patients without cirrhosis [77]. This was the basis for the current recommendation for HCC screening only in patients with NAFLD cirrhosis since the incidence of HCC is too low to justify the introduction of a screening programme to all patients with NAFLD [77, 78]. Ultrasound is a preferable method for HCC screening due to its availability and noninvasiveness, and it should be performed every six months. It has a sensitivity of 84% and a specificity of 91% for the detection of HCC. Since the majority of HCCs appear in cirrhotic livers, with a coarse echostructure and nodular appearance, the detection of early stage HCC has a lower sensitivity of only 47% [79]. Therefore, screening for HCC should be performed by experienced operators with high-quality ultrasound machines.

## 6. Conclusions

Nonalcoholic fatty liver disease is becoming the most prevalent liver disease worldwide, mostly due to its association with epidemics of overweight/obesity, T2DM, and MetS. Around 20–30% of patients with NAFLD are under risk of developing progressive liver fibrosis, which is the most important predictor of both liver-related and overall morbidity and mortality. Contrary to previous beliefs, no significant association has been demonstrated between the inflammatory component of NAFLD, i.e., NASH, and the adverse clinical outcomes. Older age (>50 years) and presence of T2DM, in addition to some genetic variants, are indicators of increased risk of having liver fibrosis. However, critical driving force for the progression of fibrosis and risk factors for this have still not been fully elucidated. Apart from the genetic profile, gut dysbiosis, weight gain, worsening of insulin resistance, and worsening of liver steatosis represent candidate factors associated with unfavourable development of liver disease. Cardiovascular events and extrahepatic malignancies, followed by liver-related deaths, represent the leading causes of mortality in NAFLD. As patients with advanced fibrosis are under highest risk of adverse clinical outcomes, efforts should be made to recognize individuals under risk and rule out the presence of this stage of fibrosis, preferably by using simple noninvasive tools. This process should start at the primary care level by using validated biochemical tests, followed by direct serum tests for fibrosis or TE in the remaining patients. Patients with advanced fibrosis should be referred to hepatologists for aggressive lifestyle modification and correction of the components of MetS, and cirrhotic patients should be screened for HCC and oesophageal varices.
